# Valorization of Co-Products from Barbecue Sauce Production Through Fermentation Processes

**DOI:** 10.3390/foods15081275

**Published:** 2026-04-08

**Authors:** Ana Catarina Costa, Joana Braga, Miguel Figueiredo Nascimento, Anabela Raymundo, Catarina Prista

**Affiliations:** 1 LEAF—Linking Landscape, Environment, Agriculture, and Food Research Center, Associated Laboratory TERRA, Instituto Superior de Agronomia, Universidade de Lisboa, Tapada da Ajuda, 1349-017 Lisboa, Portugal; acatarinacosta@isa.ulisboa.pt (A.C.C.); miguel.nascimento@casamg.pt (M.F.N.); anabraymundo@isa.ulisboa.pt (A.R.); 2 Mendes Gonçalves, Zona Industrial, lote 6, 2154-909 Golegã, Portugal

**Keywords:** barbecue sauce, bio-valorization, circular economy, fermentation, waste stream

## Abstract

Industrial food processing generates substantial byproducts, resulting in environmental challenges and economic losses. This study explores the biovalorization of sugar-rich barbecue sauce waste streams through fermentation to create value-added ingredients for sauce production and promote circular economy practices. The barbecue stream was diluted with water at 25 and 50% incorporation levels and fermented at room temperature for 12 days using a microbial consortium comprising three lactic acid bacteria (*Lactiplantibacillus plantarum*, *Lacticaseibacillus rhamnosus*, and *Weissella confusa*) and one yeast (*Saccharomyces boulardii*). Laboratory-scale fermentation was monitored by measuring pH, total soluble solids, titratable acidity, sugar consumption, and metabolite production. The consortium demonstrated effective performance, reducing pH and TSS and increasing titratable acidity for both incorporation levels over 12 days. The fermented samples were characterized by their antioxidant capacity, color, protein content, humidity, and viscosity. The total phenolic content and antioxidant activity (DPPH) increased significantly (*p* < 0.05), and the viscosity increased by 254.3% and 48.3% for the fermented streams with 25% and 50% incorporation, respectively. Antimicrobial assays revealed that the fermented samples inhibited typical spoilage bacteria and yeast. This work highlights the potential of fermentation to upcycle barbecue waste, with antimicrobial characteristics contributing to extended shelf life, sustainable food production, and circular economic practices.

## 1. Introduction

In response to consumer demand for quality and innovative products, the food industry has focused on developing superior offerings, resulting in a diverse range of products [1]. However, product variety expansion incurs significant environmental costs [2]. Increased processing, driven by consumer demand, has led to substantial waste generation within the food industry [3], making food waste a persistent global issue.

Increasing awareness of the environmental impact of waste generated by the food industry, coupled with consumers’ heightened sensitivity to ecological concerns, has generated both economic and social incentives for industries to manage waste more effectively, thereby encouraging the adoption of sustainable practices [3]. In recent years, the concept of a circular bioeconomy has emerged as a model for sustainable development, advocating for the efficient utilization of renewable biological resources [4].

The circular bioeconomy represents an integrated framework that combines the principles of circular and bioeconomies, facilitating the transition of production and consumption systems towards sustainability by enabling multiple reuse cycles of biological materials and promoting environmental stewardship [5,6]. This approach seeks to minimize waste and environmental impacts while supporting economic development by creating a system that recycles and regenerates biological materials [7,8].

Such a system is pertinent to the agricultural and food industries, where substantial volumes of byproducts and waste streams present environmental challenges and opportunities for resource recovery [9]. Advancements in waste management are pursuing solutions that valorize food residue (FR) products for end-use applications. The composition of raw materials provides macronutrients, micronutrients, vitamins, and bioactive compounds [10]. This value can be harnessed by converting FR into value-added products, optimizing company resources, and promoting circularity [11].

Food fermentation is a key biotechnological strategy for food waste recovery and an alternative to landfill disposal [12,13]. Microbial activity transforms biomass into high-value products, including organic acids, bioactive peptides, enzymes, biofuels, bioethanol, and functional ingredients, thereby reducing waste and enhancing resource circulation [11]. Integrating fermentation technologies into food waste management supports sustainable food systems, mitigating food loss and environmental impacts while facilitating the transition to a sustainable economy [14,15].

The utilization of sauces and condiments holds significant historical and cultural relevance globally [16]. Nevertheless, industrial production processes often result in waste streams when products fail to meet the specified criteria for the intended product, leading to their disposal or use as fuel, thereby incurring additional environmental and economic costs [17]. Barbecue sauce has transitioned from simple regional recipes to widely produced condiments on an industrial scale.

The global market for sauces and condiments has demonstrated consistent growth, primarily driven by evolving consumer preferences for convenience, sensory appeal, and health consciousness, with barbecue (BBQ) sauces emerging as a dynamic subcategory [18]. However, product development within this segment is increasingly shaped by sustainability concerns, including the demand for ‘clean label’ formulations, reduced sugar content, and the utilization of environmentally responsible ingredients and processing practices [18]. These market dynamics underscore the growing importance of implementing strategies for managing waste streams, presenting opportunities for innovation in industrial processes and the development of new products.

Casa Mendes Gonçalves has been proactive in reducing the impact of waste production through various strategies, including landfill disposal, destruction, and valorization for diverse end uses.

This study explores fermentation as a viable strategy for upcycling barbecue sauce waste streams. This approach involves valorization through fermentation, with the potential to produce valuable products for integration into target sauces, as fermentation can enhance flavor profiles, reduce sugar content, and impart antimicrobial properties. This study suggests that this method could reduce industrial waste and foster innovation in product development.

## 2. Materials and Methods

### 2.1. Strains

The lactic acid bacteria (LAB) strains used in this study were *Lactiplantibacillus plantarum* DSM 20205, *Lacticaseibacillus rhamnosus* DSM 20021, and *Weissella confusa* DSM 20196. These strains were consistently maintained on solid De Man, Rogosa, and Sharpe (MRS) agar (*Biokar, Allonne, France*). The yeast strain employed was *Saccharomyces boulardii* ATCC MYA-796, which was regularly preserved on solid YPD medium (containing 0.5% yeast extract, 1% peptone, 2% glucose, and 2% agar). Cells were incubated at 28 °C until visible colony growth was observed and then stored at 4 °C until use, for a maximum of one week. In the experimental trials, the fermentation consortium consisted of *Saccharomyces boulardii* (I) and a mixture of three lactic acid bacteria (C3), referred to as I + C3. For antimicrobial tests, food contaminants were obtained from the Instituto Superior de Agronomia, Yeast (ISA), and Bacteria (BISA) library, and DMSZ collection (Table 1). Cells were incubated at 28 °C until visible colony growth was achieved and then stored at 4 °C until use, for a maximum of one week.

### 2.2. Inoculum Preparation

A consortium of cell strains was prepared from cultures grown in liquid. The cells were harvested by centrifugation at 3220× *g* for 5 min, washed, and resuspended in sterile demineralized water to achieve a cell suspension of 10^9^ cells/mL. A mixture of four strains was prepared at a final concentration of 10^6^ cells/mL per strain in each flask. The glass jars were then sealed, labeled, and incubated at room temperature (22 ± 1 °C).

### 2.3. Fermentation Assays

The barbecue sauce stream (BBQ) was diluted with sterile distilled water at a 1:4 ratio, resulting in a 25% (*w*/*v*) concentration (BBQ25), and at a 1:2 ratio, resulting in a 50% concentration (BBQ50). Fermentation on a laboratory scale was conducted in 400 mL jars, with each fermentation performed in triplicate. The consortium was inoculated into each jar at a final concentration of 10^6^ cells/mL for each strain. The flasks were sealed, labeled, and incubated at room temperature (22 ± 1 °C). Samples were collected periodically until day 12.

### 2.4. Physicochemical Parameters, Substrate Consumption, and Metabolite Production

#### 2.4.1. pH, Total Soluble Solids, and Titratable Acidity

pH was measured in triplicate using a pH Go Direct Electrode Amplifier (*Vernier, Beaverton, OR, USA*), linked to LabQuest3 (*Vernier, Beaverton, OR, USA*). Total soluble solids (TSSs) were analyzed in triplicate at various fermentation times using a digital refractometer (*Hanna HI96801; Hanna Instruments, Woonsocket, RI, USA*).

The titratable acidity was analyzed using 0.5 mL of the sample diluted 1:100 with 50 mL of demineralized water. The diluted solution was titrated with a 0.1 N solution of sodium hydroxide (NaOH, *Merck KGaA, Darmstadt, Germany*) using a pH electrode (*Broadley Jones Corporation, Irvine, CA, USA*) linked to a potentiometer (pHM92 Lab pH Meter, *Radiometer, Copenhagen, Denmark*) until a pH of 8.15 was reached. Titratable acidity was expressed in grams of lactic acid equivalents per 100 g of fresh biomass using Equation (1).

(1) $$Titratable\;acidity\;(\%\;lactic\;acid)=\frac{Volume\;of\;NaOH\times \left[NaOH\right]\times equivalent\;mol\;in\;grams\;of\;lactic\;acid}{\;samples\;mass\times 10}$$

#### 2.4.2. Substrate and Metabolite Content

Sugar consumption, organic acid, and ethanol production during fermentation were analyzed using high-performance liquid chromatography (HPLC; *Hitachi, Ibaraki, Japan*). Samples were diluted 1:10 with 50 mM sulfuric acid (H_2_SO_4_, *BDH, Dubai, United Arab Emirates*) and centrifuged at 4 °C (3220× *g*, 15 min). The supernatant was collected, diluted 1:2 with the same acid, and directly filtered into the sampling tubes using a nylon membrane with a 0.22-μm pore diameter.

Calibration curves were prepared for sugars, glycerol (0–4 g/L), organic acids (0–2 g/L), and ethanol (0–2.5% *v*/*v*). Samples were analyzed at 65 °C using high-performance liquid chromatography (HPLC, *Hitachi, Ibaraki, Japan*), with an ion-exclusion column 5310, (*RezexTM ROA Organic Acid H+ (8%) column, 300 × 7.8 mm, Phenomenex, Torrance, CA, USA*), linked to an autosampler 5260, and injection pump 5160, and connected to a refractive index detector 5450 for sugars, glycerol, and ethanol, and a UV-VIS 5420 detector for organic acids. Samples (20 μL) were injected, and 5 mM sulfuric acid (H_2_SO_4_, *BDH, Dubai, United Arab Emirates*) was used as the mobile phase at a flow rate of 0.5 mL/min.

### 2.5. Nutritional Composition

#### 2.5.1. Total Protein Content

Protein content was determined using the DUMAS method with an NDA 702 dual-carrier gas nitrogen analyzer (*VELP Scientifica, Italy*). Samples (150 mg, in triplicate) were mixed with 33–62 mg of superabsorbent powder (*VELP Scientifica, Italy*) to form a paste, which was combusted at high temperatures in an oxygen-rich environment, converting nitrogen to nitrogen gas (N_2_). Nitrogen content was determined using a thermal conductivity detector and converted to protein content using a general conversion factor of 6.25 [19].

#### 2.5.2. Humidity Content

To determine the humidity of the samples, 2 g of each sample was weighed in pre-weighted aluminum foils and dried in a stove at 105 °C for 3 days until the weight stabilized [20]. The humidity percentage was calculated as the difference between the wet and dry samples. Triplicate measurements were performed for each sample.

### 2.6. Determination of Antioxidant Activity and Phenolic Content

#### 2.6.1. Sample Preparation and Extraction

Samples for antioxidant capacity (DPPH, FRAP, and TPC) were prepared by centrifuging 5 *g* of the sample at 4 °C (3220× *g*, 15 min at 4 °C) to obtain a clear supernatant. The supernatant was collected, filtered through a 0.45 μm pore diameter acetate cellulose membrane, placed in 10 mL Falcon tubes, and used for chemical analysis.

For the TPC, an alcoholic extract was used to avoid potential interference from small peptides and amino acids in the measured absorbance values [21]. Samples were weighed to 1 g in a 15 mL Falcon tube, 9 mL of 96% ethanol (C_2_H_6_O, *Aga, Lisbon, Portugal*) was added (1:10 *w*/*v*), and the mixture was shaken for 12 h at room temperature [22]. Finally, the samples were centrifuged at 3220× *g* for 15 min at 4 °C, and the supernatants were collected.

In addition to phenolic compounds, the antioxidant capacity (measured by DPPH, FRAP, and ABTS^+^ assays) of the fermented product may also be contributed by several water-soluble molecules, namely amino acids, peptides, and exopolysaccharides, produced by the consortium microorganisms. Therefore, the antioxidant capacity of BBQ products was measured directly in the centrifuged sample, as Liu et al. (2018) did for fermented tomato juice [23].

#### 2.6.2. ABTS^+^

Total antioxidant activity was analyzed using the ABTS (*2,2′-azino-bis(3-ethylbenzothiazoline-6-sulfonic acid*) radical cation) method [24]. The ABTS 7 mM solution (C_18_H_18_N_4_O_6_S_4_, *Sigma, St. Louis, MO, USA)* reacted with the potassium persulfate 140 mM solution *(K_2_S_2_O_8_, Sigma, St. Louis, MO, USA)* and was left in the dark for 16 h. Then, it was diluted with 96% ethanol (C_2_H_6_O, *Aga, Lisbon, Portugal*) to an absorbance of 0.70 ± 0.05 at 734 nm. In a microplate, 3 μL of sample supernatant from the prepared samples was mixed with 300 μL of diluted ABTS^+^ solution, stirred, and left in the dark for 6 min. The absorbance was read at 734 nm in a microplate spectrophotometer (*Mobi, μ2 Microdigital Co., Ltd., Seongnam-si,* *Republic of Korea*) using 96% ethanol as the blank. The calibration curve (0–200 μg/mL) was expressed as Trolox equivalent (*Sigma, St. Louis, MO, USA*).

#### 2.6.3. DPPH Radical Scavenging Assay

The DPPH (*2,2-diphenyl-1-picrylhydrazyl*) (C_18_H_12_N_5_O_6_, *TCI, Tokyo, Japan*) free radical-scavenging assay was performed according to the method described by Liu et al. [23], with some modifications. The DPPH 103.5 μM solution was prepared in methanol (CH_3_OH, *Merck KGaA, Darmstadt, Germany*). In a microplate, 8.5 μL of each sample and 331.5 μL of DPPH solution were added in triplicate. The microplate was left in the dark for 45 min, and then the absorbance was read at 515 nm (*Mobi, μ2 Microdigital Co., Ltd., Republic of Korea*). The blank of the method was water. A calibration curve was done in the range 0–200 μg/m Trolox (*Sigma, St. Louis, MO, USA*). The results were expressed in Trolox equivalents (TEAC)/100 g of fresh biomass.

#### 2.6.4. Ferric Reducing Ability of Plasma

Antioxidant capacity was analyzed using the FRAP method [25] with adaptations. The FRAP solution was composed of 25 mL of 0.3 M acetate buffer (C_2_H_3_NaO_2_·3H_2_O, *Merck KGaA*, *Darmstadt, Germany*), 2.5 mL of TPTZ 10 mM (*2,4,6-Tris(2-pyridyl)-s-triazine*, C_18_H_12_N_6_, *Alfa Aesar, MA, USA*) in 40 mM hydrochloric acid (HCl, *BDH, Dubai, United Arab Emirates*), and 2.5 mL of 20 mM ferric (III) chloride hexahydrate (FeCl_3_·6H_2_O, *Sigma, MO, USA*) at room temperature. In microplate wells, 10.5 μL of sample supernatant, 30.9 μL of MiliQ water, and 309 μL of FRAP reagent were mixed in triplicate and incubated at 37 °C for 30 min in the dark. Absorbance was read at 595 nm using MiliQ water as the blank (*Mobi, μ2 Microdigital Co., Ltd., Republic of Korea*). The calibration curve (0–200 μg/mL) was expressed on Trolox (*Sigma, St. Louis, MO, USA*).

#### 2.6.5. Phenolic Content

##### Total Phenolic Content (TPC)

TPC was determined using a method adapted from Swain and Hillis [26], based on a colorimetric assay with modifications. In a microplate well, 17.56 μL of the prepared sample, 16.4 μL of Folin–Ciocalteu reagent (*Sigma, St. Louis, MO, USA*), and 281 μL of MiliQ water were added and left to react for 3 min. Then, 35.13 μL of 10% (*w*/*v*) sodium carbonate (NaCO_3,_ *AnalaR, Dubai, United Arab Emirates*) was added and left to react in the dark for about 2 h. Absorbance was read at 725 nm in a microplate spectrophotometer (*Mobi, μ2 MicroDigital Co., Ltd., Republic of Korea*) using MiliQ water as the blank. The calibration line from 0 to 350 μg/mL was expressed in gallic acid (C_7_H_6_O_5_, *Sigma, St. Louis, MO, USA*).

##### Total Phenolic Index (TPI)

For this analysis [27], 1 g of the sample was diluted in 9 mL demineralized water (1:10), centrifuged at 4 °C, 3220× *g*, 15 min, and the supernatant was transferred to 10 mL Falcon tubes. The samples were measured in triplicate using a quartz cuvette (*ultra-Micro 10 mm Fo3, Hellma, Merck, Darmstadt, Germany*). Absorbance was read using spectrophotometry (*UV-visible Biochrom Libra S22, 190–1100 nm, Biochrom, Cambridge, UK*) at 280 nm. The calibration curve from 0 to 0.08 mg/mL was expressed in terms of gallic acid (C_7_H_6_O_5_, *Sigma, St. Louis, MO, USA*).

### 2.7. Color

Color was evaluated using a colorimeter (CR-400; *Konica Minolta, Tokyo, Japan*). The samples were placed in Petri dishes and measured five times to ensure accuracy. The L*, a*, and b* values of the diluted samples were analyzed before and after fermentation. L*, a*, and b* indicate lightness (0 = black, 100 = white), red (+ to green (−)), and yellow (+ to blue (−)), respectively [28], where *i* denotes the sample after fermentation and 0 denotes the sample before fermentation. The total color difference (ΔE*) between the fermented and non-fermented samples was calculated using Equation (2):

(2) $$∆E*=\sqrt{{\left(L_{1}^{*}-L_{2}^{*}\right)}^{2}+{\left(a_{1}^{*}-a_{2}^{*}\right)}^{2}+{\left(b_{1}^{*}-b_{2}^{*}\right)}^{2}}$$

### 2.8. Viscosity Analysis

For viscosity evaluation, a controlled stress rheometer (*Thermo Fisher Scientific, Waltham, MA, USA*) coupled with an Eheim professional 3 air compression system and a peltier temperature-controlled system (*Thermo Scientific Haake MARS III Controller*) at 20 ± 0.5 °C, was used.

A series of apparent viscosity tests was conducted to generate flow curves, with the samples subjected to varying shear rates (1 × 10^−5^ to 500 s^−1^) using a parallel serrated plate (PP35Ti) with a 1 mm gap (previously optimized) and a 60 mm plate diameter. The apparent viscosities of BBQ25 and BBQ50 were analyzed, and the unfermented and fermented samples were compared. Viscosity values were plotted against shear rates, and the Williamson model [29] was fitted (Equation (3)), using Origin 2019 (*OriginLab, Northampton, MA, USA*):

(3) $$\eta =\;\frac{\eta_{0}\;}{{1+(k_{\dot{\gamma }})}^{m}}$$

$\eta_{0}$ is the zero-shear Newtonian viscosity (Pa.s), *k* is the consistency coefficient (Pa.s), and *m* is the dimensionless shear-thinning index.

### 2.9. Shelf Life of the Fermented Products Produced

The shelf life of the fermented barbecue products was evaluated throughout the experimental trial to assess the stability of the fermented products obtained in this study. The pH, TSS (in °Brix), and titratable acidity were monitored at various points throughout the shelf life of the product. HPLC was used to measure metabolite content at 0 and 12 months of the fermentation process.

### 2.10. In Vitro Antimicrobial Activity 

Antimicrobial activity was tested using a drop-testing plate method to evaluate the inhibitory capacity of BBQ25- and BBQ50-fermented products against typical contaminants from Mendes Gonçalves (Table 1). The samples were pasteurized by immersion in a water bath at 85 °C for 5 min. Rich media, namely, MRS, YPD, and BHI, were prepared with double the recommended concentration and agar, and the samples were diluted 1:2 in the media with each BBQ-fermented product at 25% and 50% incorporation. Bacteria and yeast strains were grown to the mid-exponential phase and diluted (decimal) to obtain 1–2 cells/µL in 3 µL on the last dilution plates containing the medium. Plates were prepared in duplicate with two controls (MRS, YPD, and BHI only) at the start and end of the trial to ensure microbial viability. All plates were incubated at 28 °C for 3 days.

### 2.11. Statistical Treatment of Data

Means and standard deviations for the non-fermented and fermented barbecue streams were calculated for statistical analysis. The results of a *t*-test were used to compare the two variables at a 95% confidence level (α = 0.05) using Prism 11 (*GraphPad Sof85 °Ce, Boston, MA, USA*).

## 3. Results and Discussion

### 3.1. Fermentative Process Evolution of the BBQ Diluted Streams

The preliminary results obtained by the research team point to a consortium of three lactic acid bacteria (*Lactiplantibacillus plantarum*, *Lacticaseibacillus rhamnosus*, and *Weissella confusa*) and a yeast (*Saccharomyces boulardii*) as the most effective in reducing pH, achieving the same final pH and a more pronounced reduction in sugars, with the additional benefit of being uniquely recognized as a probiotic. Based on these results, this consortium was chosen to ferment the BBQ waste stream at 25% (BBQ25) and 50% (BBQ50) incorporation.

**Figure 1:**
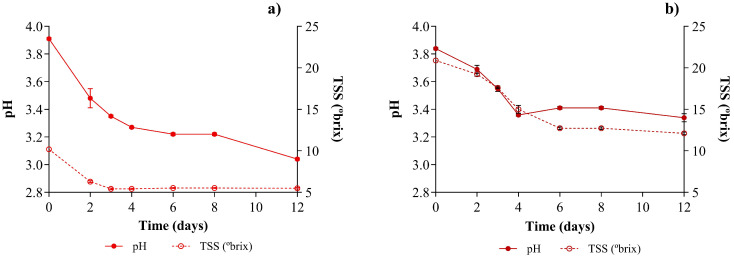
Fermentative process evolution of the fermentation with I + C3 consortium, for BBQ25 (**a**) and BBQ50 (**b**). Values are expressed as mean ± standard deviation (*n* = 3).

The evolution of the fermentative process was monitored by measuring the pH and TSS (°Brix) over 12 days of fermentation for BBQ at 25% and 50% stream incorporation. Data regarding the pH and TSS are presented in Figure 1.

Regarding BBQ25 (Figure 1a), the pH values decreased progressively until day 12, from an initial value of 3.91 to 3.04. This decrease shows the predominant metabolic activity of lactic acid bacteria. The TSS value decreased steadily during the first 2 days of fermentation, from an initial value of 10.2 °Brix to 6.29 °Brix. Afterward, TSS tended to stabilize at approximately 5.42 Brix, maintaining this value through day 12. This result is consistent with the higher initial rates of sugar consumption by lactic acid bacteria and yeasts, and the production of metabolites such as lactic acid and acetic acid [30] as reflected by the decrease in pH. As fermentation proceeds, the amount of sugar decreases, and acids accumulate, helping stabilize TSS.

In contrast to BBQ25, for BBQ50 (Figure 1b), the pH decreased more sharply from days 2 to 4 and stabilized at 3.36 for the remainder of the fermentation process. Regarding TSS, as expected, the initial value was twice that of BBQ25 (20.9 °Brix), due to the higher level of stream incorporation. This difference may lead to slower metabolic activity due to higher initial osmotic stress, as reflected in a slower initial pH decrease and a higher pH. After day 6, the TSS values for sugar consumption stabilized at a higher final value (12.1 Brix), with a behavior similar to that of BBQ25, due to the balance between sugar consumption and metabolite production.

**Figure 2:**
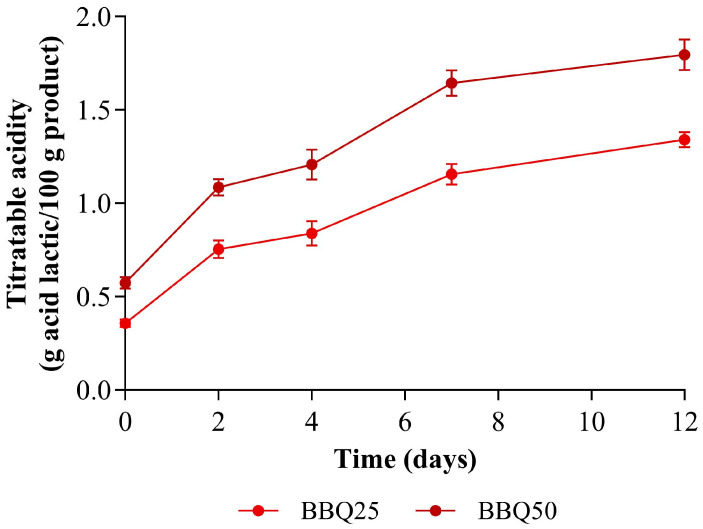
Titratable acidity of the fermentation with I + C3 consortium at both incorporation levels (25% and 50%). Values are expressed as mean ± standard deviation (*n* = 3).

The titratable acidity, expressed as lactic acid, was monitored throughout fermentation (Figure 2).

In the experimental trial with 25% stream incorporation (BBQ25), the initial value was 0.358 g lactic acid/100 g. After 12 days of fermentation, it increased to 1.341 g lactic acid/100 g, consistent with the production of lactic acid by the lactic acid bacteria of the consortium [31]. In the experimental trial with 50% incorporation of stream, there was also an increase in titratable acidity to 1.795 g lactic acid/100 g. BBQ50 has higher acidity than BBQ25; the relative increase in TA is similar at both levels of incorporation (73% and 69%, for BBQ25 and BBQ50, respectively), suggesting similar behavior.

**Figure 3:**
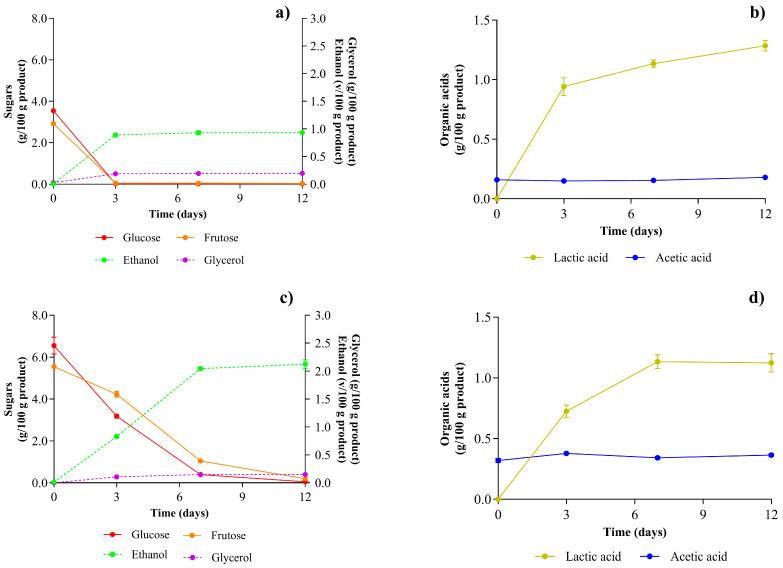
Evolution of sugars and primary metabolites along the fermentation process of the diluted BBQ steams. Values are expressed as mean ± standard deviation (*n* = 2). Concentration of the main sugars and metabolites in BBQ25 (**a**,**b**) and BBQ50 (**c**,**d**).

The consumption of sugars and production of the main fermentation metabolites were also determined by measuring their concentrations over 12 days of fermentation (Figure 3 and Table 2). As expected, the initial concentration of fermentable sugars (mainly glucose and fructose) was higher in BBQ50 (Figure 3). In addition, the concentration of acetic acid in BBQ25 was half that in BBQ50 owing to the higher dilution of the stream, which contained vinegar as an ingredient. Despite these initial differences, for both BBQ formulations, the concentration of fermentable sugars decreased during fermentation owing to the presence of *S. boulardii* and lactic acid bacteria (LAB). This reduction was more pronounced for glucose than for fructose, as expected from the glucophilic behavior of both LAB [32,33,34,35] and *S. boulardii* [36,37]. These sugars are fermented to lactic acid by LAB and to ethanol by heterofermentative LAB and *S. boulardii*. Sugar consumption was consistent with the more pronounced increase in lactic acid (and decrease in pH) and ethanol levels in BBQ25 during the first few days of fermentation, reflecting the slower metabolism observed in BBQ50. After the main hexoses are consumed, fermentation is stabilized in BBQ-fermented products.

Overall, the organic acid profile indicates that BBQ waste stream fermentation was mainly driven by lactic and alcoholic metabolism, with specific pathways for individual acids [38]. The significant accumulation of lactic acid likely results not only from sugar fermentation by *Lactiplantibacillus plantarum* and *Lacticaseibacillus rhamnosus* but also from the probable conversion of malic acid into lactic acid through malolactic activity [39]. Additionally, *Weissella confusa* is heterofermentative, but in fructose-containing systems, it may channel metabolism toward ethanol rather than acetate; this aligns with reports that *Weissella confusa* can produce ethanol but not mannitol in the presence of fructose [40]. This view aligns with the minimal and statistically non-significant changes observed in acetic acid concentration, despite clear sugar depletion and notable increases in ethanol and glycerol, also highlighting active fermentation by *Saccharomyces boulardii*.

### 3.2. Effect of Fermentation on the Chemical Composition of the Diluted BBQ Streams

#### 3.2.1. Protein Composition and Humidity

The total protein content and humidity levels were analyzed in BBQ25 and BBQ50 before (NF) and after fermentation (F) to evaluate the effect of the process on the nutritional composition of each diluted stream (Table 3).

The observed increase in moisture content and decrease in protein content in the fermented samples, with a significant difference (*p* < 0.05), can be attributed to fermentation driven by microbial metabolism and structural changes in the matrix. The conversion of fermentable sugars into metabolites (Figure 3 and Table 2) occurs in the aqueous phase, likely increasing the relative water content of the product [41]. Concurrently, the increase in viscosity (see Section 3.3.2) suggests that the production of exopolysaccharides by LAB may enhance water retention and contribute to the higher moisture content of the fermentable samples [42].

Protein was measured using the Dumas combustion method, which converts total nitrogen, and a standard conversion factor (6.25) was used to calculate the protein content. As the water fraction of the sample increased, the percentage of protein based on the fresh product weight decreased. Research on tomato-based fermented products has shown that lactic acid fermentation changes their physicochemical properties, which can influence hydration and nutrient distribution [43,44,45], such as water and protein content, confirming a dilution effect [41].

#### 3.2.2. Phenolic Content and Antioxidant Capacity

Considering that BBQ sauce contains several compounds that may contribute to the antioxidant capacity of the streams, such as peptides, exopolysaccharides, flavonoids, and phenolic acids derived from tomatoes [46], spices [47], and microbial metabolism [48], the total phenolic content (TPC) and antioxidant capacity were evaluated in the fermented streams and compared with those in the non-fermented streams.

As expected, the TPC was considerably higher in BBQ50 than in BBQ25 non-fermented, primarily because of the higher dilution level of the BBQ25 stream, although both fell within the range usually found for this type of tomato-derived sauce [49].

The values obtained for the stream before (NF) and after fermentation (F) showed a statistically significant (*p* < 0.05) increase in phenolic compounds in both fermented samples (Table 4). Although for BBQ50 a larger increase from 37.617 mg GAE/100 g to 59.408 mg GAE/100 g was observed, the relative increase was greater in BBQ25 (from 13.742 mg GAE/100 g to 26.683 mg GAE/100 g, corresponding to 94% of the initial value).

Regarding the soluble phenolic content (TPI), fermentation increased the TPI for BBQ50 and BBQ25 from 16.989 mg GAE/100 g to 17.382 mg GAE/100 g and from 9.644 mg GAE/100 g to 10.887 mg GAE/100 g, respectively, although the increase for BBQ50 was very small. This increase is consistent with the observations for TPC, reinforcing the idea that fermentation may enhance the solubility of phenolic compounds [23,45,50].

Three assays were used to evaluate antioxidant power: DPPH, FRAP, and ABTS^+^. These methods evaluate the capacity of an antioxidant to reduce different oxidants (*2,2-diphenyl-1-picrylhydrazyl; 2,4,6- tri(2-pyradyl)-1,3,5-triazine* (reduction of Fe^3+^ to Fe^2+^); and *2,2′-azino-bis(3-ethylbenzothiazoline-6-sulfonic acid)*, which change color upon reduction [51].

Overall, there was a general increase in antioxidant capacity for all methods applied to the fermented streams (Table 5). However, only BBQ25 showed significant differences (*p* < 0.05) between the non-fermented (NF) and fermented (F) samples. Although the remaining samples did not appear to be significantly different, this increase indicates that fermentation enhances antioxidant capacity [52,53].

The lack of significant differences in FRAP and ABTS after fermentation should not be viewed as a decrease in antioxidant activity. Instead, it emphasizes the different principles behind each test [54,55]. FRAP measures ferric reducing ability through an electron transfer process, while ABTS evaluates a broad radical-scavenging capacity in both water-based and organic solvents. DPPH is often more sensitive to specific radical-scavenging compounds [54,55]. Therefore, the increase in DPPH, especially in BBQ25, indicates that fermentation promoted the release and/or transformation of compounds with higher radical-scavenging activity, even though the overall reducing power measured by FRAP remained unchanged. Additionally, the final acidic pH of the fermented products may have limited the response of some phenolic antioxidants, particularly in electron-transfer systems, contributing to the modest changes observed in FRAP and ABTS^+^ [56,57].

LAB respond quickly and effectively to free radicals by enhancing antioxidant activity, chelating metal ions, producing antioxidant enzymes, and producing other metabolites with oxidant capacity, thus mitigating damage caused by oxidative stress [53,58]. In addition, biotransformation processes that increase the antioxidant capacity of fermented foods and beverages are also well known in fermentations by LAB and yeast [53,58].

Tomatoes contain high levels of phenolic acids, with ferulic, caffeic, and p-coumaric acids being the most abundant [46,59]. In vegetables and fruits, many of these compounds are bound to plant cell wall components, such as cellulose, hemicellulose, and pectin, through glycosidic or ester bonds, and enzymatic action is required to release them, thereby increasing their bioavailability. Fermentation by LAB and yeast is known for their ability to break down complex compounds and yield them in a more accessible form [60]. These microorganisms synthesize hydrolytic and oxidative enzymes, including esterase, decarboxylase, and phenolic acid reductase, which release and alter phenolic acids, such as ferulic, p-coumaric, caffeic, and gallic acids from plant cell wall matrices [23,45,61]. For example, free phenolic acids are released from glycosylated hydroxycinnamic acids in plants (esterified) by *Lactiplactibacillus plantarum* [62] and *Lacticaseibacillus rhamnosus* [52,53], as well as by *Weissella confusa* [63].

In addition to the enzymatic transformation of phenolic compounds, other molecules, such as EPS produced by LAB species, including those used in the present study, can contribute to an increase in the antioxidant capacity of fermented products [64,65,66]. These compounds contribute to the increase in antioxidant power of the fermented products through their action as free-radical, hydroxyl, and superoxide radical scavengers due to the hydrolysis of these biological molecules in an acidic environment through mechanisms involving hemiacetal esters (HAEs) [67]. These active substances provide electrons to free radicals, which become stable forms, and eventually reduce the concentration of free radicals [64,66,68]. Also, an antioxidant effect was observed for *Lb. plantarum*, which EPS inhibit H_2_O_2_ induced apoptosis [69].

Considering that our fermentation was performed using a consortium with *Lb. plantarum*, *Lb. rhamnosus*, and *W. confusa*, which are known to produce both hydrolytic and oxidative enzymes and EPS, it is possible to hypothesize that mechanisms involving enzymatic activity and EPS production may be responsible for the increases in TPC and TPI, together with the increase observed in antioxidant capacity. A similar result was reported by Li et al. (2014) [48] and Sungatullina et al. (2023) [70] in milk products, as well as by Lui et al. (2018) in fermented tomato juice [23]. Interestingly, our results also indicate a stronger effect of fermentation in BBQ25, which is consistent with the lower level of EPS at 50% waste-stream incorporation (see Section 3.3.2) and with lower enzymatic activity under the stronger osmotic stress conditions found in BBQ50.

### 3.3. Effect of Fermentation on Color and Physical Properties of the Diluted Streams

#### 3.3.1. Color Parameters

Color analysis of the barbecue streams was performed to characterize them. Sample color analysis was measured using color space parameters: L*, a*, and b* (Table 6).

Regarding the darkness of the sample, as measured by its L* parameter, it was observed that for BBQ50, there was a significant (*p* < 0.05) increase in lightness. However, this result suggests that increasing the antioxidant capacity in fermented samples could reduce oxidation and prevent sample darkening [71,72]. In contrast, for BBQ25, the opposite trend was observed, with a significant (*p* < 0.05) decrease in lightness.

**Table 6:** Color analysis of the barbecue stream and the barbecue diluted before and after fermentation. Values are expressed as the mean ± standard deviation (*n* = 5). Different roman numbers and letters indicate significant differences (*p* < 0.05) between non-fermented (NF) and fermented (F) samples for each concentration, according to the *t*-test.

Color					
Samples		L*	a*	b*	ΔE
BBQ25	NF	45.192 ± 3.174 ^I^	14.316 ± 1.141 ^IV^	42.818 ± 1.833 ^V^	21.559
	F	31.706 ± 1.743 ^II^	16.250 ± 0.449 ^III^	26.110 ± 2.628 ^VI^	
BBQ50	NF	29.138 ± 1.618 ^b^	21.942 ± 0.645 ^c^	25.320 ± 2.890 ^e^	12.851
	F	36.468 ± 2.747 ^a^	21.782 ± 0.766 ^c^	35.874 ± 3.554 ^d^	

For the a* parameter, which indicates redness, the BBQ50 sample has a higher value before fermentation, suggesting a more pronounced red hue that remains constant after fermentation, with no significant differences (*p* > 0.05). BBQ25 had a more pronounced red hue after fermentation. Lastly, the b* parameter, which indicates a yellow-blue balance, showed an increase in BBQ50, suggesting a consistent yellow hue after fermentation, whereas in BBQ25, there was a decrease in the yellow hue. These changes were significantly different (*p* < 0.05).

Color differences perceivable by the human eye were analyzed using the total color difference (ΔE) parameter, which indicated that when the value exceeded 5 [73], the color difference was perceptible, suggesting that the color differences before and after fermentation for BBQ50 and BBQ25 were noticeable to the human eye and more pronounced for BBQ25.

#### 3.3.2. Apparent Viscosity

**Figure 4:**
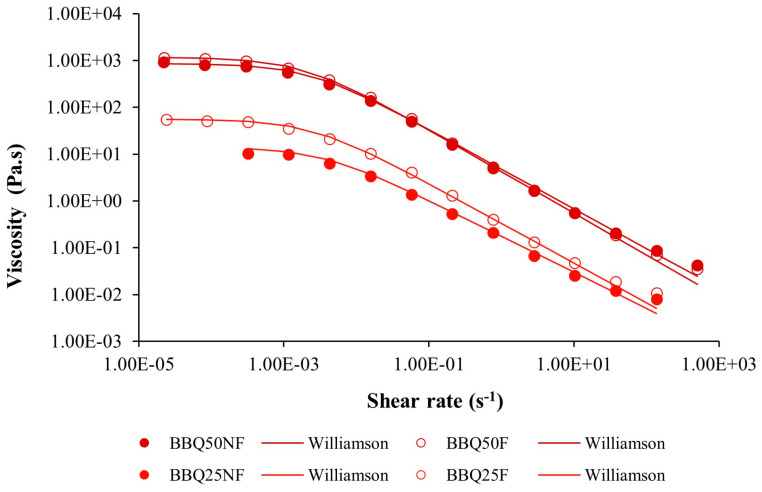
Apparent viscosity of barbecue diluted at 25% and 50%, before and after fermentation.

The apparent viscosity before and after fermentation was evaluated to verify whether significant changes occurred due to microbial metabolism (Figure 4). The fermented samples exhibited non-Newtonian behavior, characterized by two stages in the graph: (i) at low shear rates, the viscosity remained almost constant (zero-shear viscosity (η_0_)), and (ii) as the shear rate increased, the viscosity began to decrease.

The fitted parameter values from the Williamson model are presented in Table 7 to better understand the effect of fermentation on the apparent viscosity.

**Table 7:** Parameters obtained from the Williamson model for barbecue diluted at 25% and 50% before and after fermentation. Values are expressed as the mean ± standard deviation (*n* = 3). Different letters indicate significant differences (*p* < 0.05) between non-fermented (NF) and fermented (F) samples for each concentration, according to the *t*-test.

Samples		η_0_ (Pa.s)	*k* (Pa.s)	*m*	R^2^
BBQ25	NF	12.72 ± 1.42 ^b^	229.85 ± 85.04	0.773 ± 0.035	0.999 ± 0.001
	F	45.08 ± 9.86 ^a^	385.66 ± 35.13	0.830 ± 0.017	0.996 ± 0.003
BBQ50	NF	730.50 ± 115.60 ^B^	352.95 ± 82.65	0.863 ± 0.023	0.999 ± 0.009
	F	1083.68 ± 73.21 ^A^	505.30 ± 58.15	0.897 ± 0.012	0.999 ± 0.001

For BBQ-fermented products, there was a significant increase (*p* < 0.05) in zero-shear viscosity after fermentation. BBQ25 showed a higher increase in limit viscosity with fermentation, likely because of the higher production of compounds that increase viscosity, such as exopolysaccharides, which confer viscosity to the sample [74,75].

Regarding the *k* values (Table 7), the consistency parameter was higher for BBQ-fermented products (BBQ25 and BBQ50) than for non-fermented products. A similar pattern was observed for the *m* parameter with increases in BBQ25 and BBQ50.

In recent years, EPS production has been reported in LAB, especially by *Fructilactobacillus*, *Lacticaseibacillus, Lactiplantibacillus*, *Lactobacillus*, *Lactococcus*, *Latilactobacillus*, *Lentilactobacillus*, *Leuconostoc*, *Limosilactobacillus*, *Pediococcus*, *Streptococcus*, and *Weissella* [76,77]. Among the EPS-producing species used in the present study were *Lacticaseibacillus rhamnosus* [78], *Lactoplantibacillus plantarum* [79], and *Weissella confusa* [80].

As reported by several authors, LAB species produce EPS under stressful conditions as a strategy to protect themselves from environmental stresses and enhance their adaptability and survival [81,82,83]. EPSs form a protective polymer layer around microbial cells, which is particularly important under harsh conditions. Importantly, they protect microbial cells against desiccation, temperature extremes, pH fluctuations, oxidative damage, and osmotic stress by forming a high-water-content polysaccharide layer around the cell surface, thereby facilitating adhesion, enhancing resistance to environmental conditions, and improving stress resistance, whereas their anionic properties help capture essential minerals and nutrients [84].

The fermentation of BBQ waste streams poses several environmental stresses to the fermentative LAB strains used as starters, such as low pH (the initial pH is already quite low due to the addition of acetic acid to BBQ formulations), the presence of acetic acid, osmotic stress, and depletion of nutrients, which have been pointed out as factors against which the production of EPS is an important protective mechanism of LAB.

In the present study, although EPS production was not quantified, the production of these compounds was primarily assessed by visually observing the slimy and ropy phenotype of the colonies in medium containing solidified BBQ25 and BBQ50, which allowed us to state that these polysaccharides were produced by the three LAB strains under the conditions used, as previously reported by Jurášková et al. (2022) [65] and He et al. (2025) [78]. Curiously, and in accordance with our later results, this phenotype was more evident in the medium with BBQ25, suggesting higher EPS production in the presence of 25% of the waste BBQ stream.

The production of microbial EPSs varies depending on the species and growth conditions and is influenced by environmental changes that modulate enzyme activity (inhibition or stimulation) and protein synthesis (induction or repression), such as pH, temperature, dissolved oxygen concentration, solute concentration, or salinity [65,82,85,86]. The fact that BBQ25 showed a significantly higher relative increase in viscosity (254.3%) than BBQ50 (48.3%) indicates a phenotype well correlated with higher EPS production under mild osmotic stress conditions. A similar result was observed by He et al. (2025) when studying the correlation between EPS production and osmotic saline stress resistance *in Lb. rhamnosus*, reporting an increase in EPS yield with increasing sodium lactate concentrations up to 0.4 mol/L sodium lactate, followed by a decrease for sodium lactate concentrations of 0.6 mol/L or higher. This result was correlated with the inability of *Lb. rhamnosus* to tolerate the hyperosmotic environment leading to a disruption in cellular metabolic pathways and a reduction in viable counts of *Lb. rhamnosus*. This EPS synthesis response pattern induced by mild osmotic stress was also observed by Lakra et al. (2020) in *W. confusa* [87] and by Nguyen et al. (2021) [79] in *Lb. plantarum*.

In the same direction, other results reinforce the idea of higher EPS production in BBQ25, such as the higher increase in the percentage of humidity of the fermented product also observed in cheese fermented by *Lb. plantarum* EPS-producing strains [88], and the higher relative increase in antioxidant capacity obtained in BBQ25 (see Table 5), which can also be attributed to the antioxidant role of EPS produced by different species of LAB [64,65].

### 3.4. Shelf Life of the Fermented Streams

**Figure 5:**
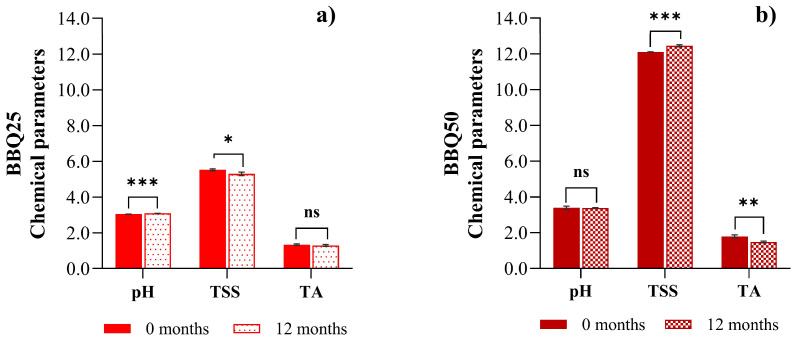
Shelf-life evolution of pH, TSSs and Titratable acidity (T.A, express in g of acid lactic per/100 g of fresh biomass) in the fermented BBQ25 (**a**) and BBQ50 (**b**). Values are expressed as mean ± standard deviation (*n* = 3) If *p* < 0.05, it is marked as (*); if the *p* > 0.01, it is marked as (**); if the *p* < 0.001, it is marked as (***) and if the value is not significant it is marked as (ns).

The shelf life of the fermented samples was monitored for over 12 months with respect to pH, TSSs (°Brix), and titratable acidity. The results obtained for BBQ25 and BBQ50 after 12 months of storage at room temperature are shown in Figure 5.

In both formulations, over one year, the pH and TSS remained constant, with no significant fluctuations. In BBQ25, the pH values remained consistent at 3.00–3.10, while TSS was 5.5 °Brix. The BBQ50 pH values remained at 3.30–3.50 and TSS at 11–12 °Brix.

Regarding titratable acidity, Figure 5 show the data obtained at the beginning and after 1 year of room-temperature preservation for BBQ25 and BBQ50, respectively. Only minor, non-significant changes were observed during this period in lactic and acetic acids, reflecting the decreased viability of lactic acid bacteria. Despite the verified presence of lactic and acetic acids, the decrease in titratable acidity in BBQ50 indicates that metabolic activity remains. This decrease suggests that metabolic transformations of the main organic acid, probably related to the secondary metabolism of LAB and yeast, may occur.

**Figure 6:**
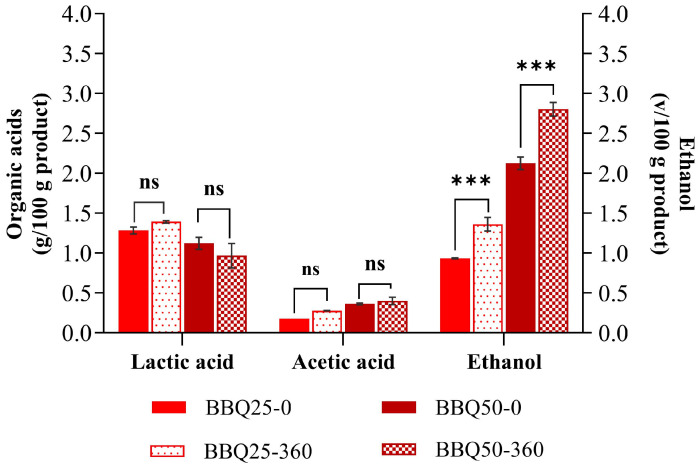
Evolution of lactic acid, acetic acid and ethanol concentration between the end of fermentation and after 360 for fermented BBQ25 and BBQ50. Values are expressed as mean ± standard deviation (*n* = 3) If the *p* < 0.001, it is marked as (***) and if the value is not significant it is marked as (ns).

In contrast, a significant increase in ethanol was observed in BBQ-fermented products (Figure 6), resulting from the presence of viable yeast cells. Given that the products were not pasteurized and were stored at room temperature, this increase is not concerning, as it enhances product preservation, suggesting that both formulations are likely to maintain their characteristics throughout the product’s shelf life.

### 3.5. Antimicrobial Activity

Antimicrobial activity was evaluated to determine the potential of the fermented barbecue stream to inhibit the growth of specific microorganisms. These microorganisms were chosen among those usually found in spoiled fruit-containing sauces: two LAB (*Lentilactobacillus parabuchneri* and *Levilactobacillus brevis*) and four fructophilic yeasts (*Zygosaccharomyces bailii*, *Zygosaccharomyces rouxii*, *Pichia manshurica,* and *Starmerella stellata*). In addition, the resistant endospore-forming species *Bacillus cereus* was tested.

**Figure 7:**
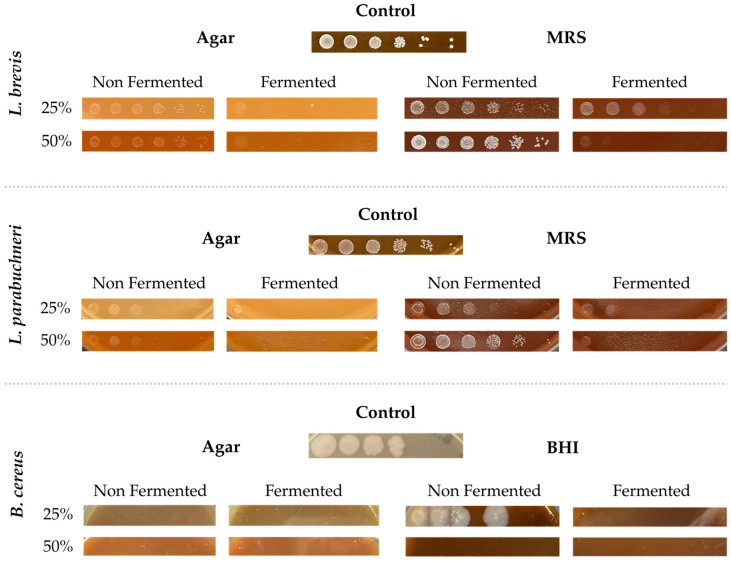
Drop test analysis of microbial growth for bacteria (*L.parabuchneri*, *L. brevis* and *B. cereus*) on agar and MRS/BHI in the barbecue fermented samples at 25% and 50% of incorporation.

**Figure 8:**
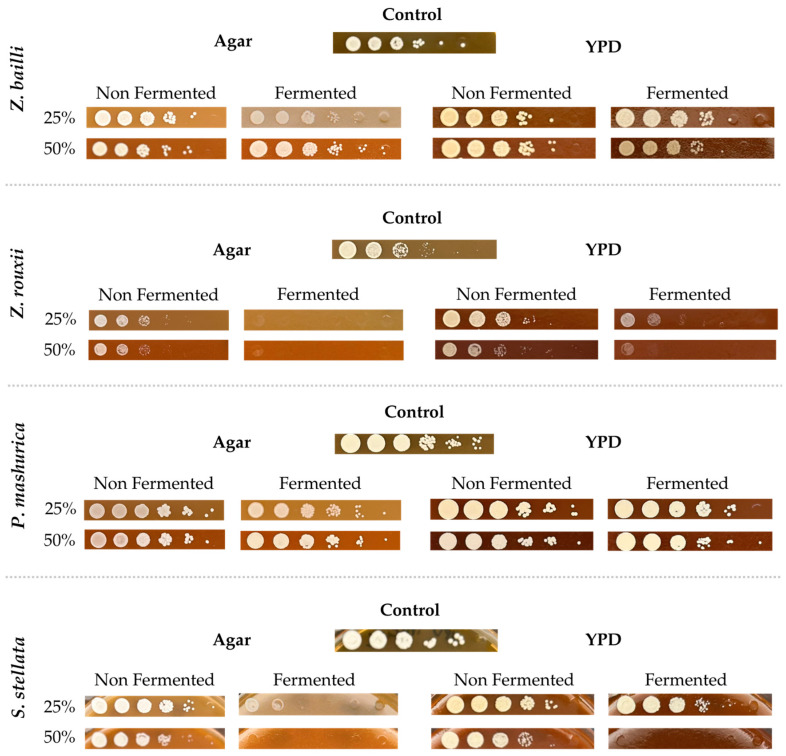
Drop test analysis of microbial growth for Yeasts (*Z. bailii*, *Z. rouxii*, *P. mashurica*, and *S. stellata*) on agar and YPD in the barbecue fermented samples at 25% and 50% of incorporation.

Figure 7 and Figure 8 show the results obtained for the bacteria and yeast tested, respectively. Images were captured after three days of incubation to allow microbial growth. Solid rich media with each fermented stream incorporated at a 1:2 proportion was used for the yeast (YPD), lactic acid bacteria (MRS), and B. cereus (BHI) to provide sufficient nutrients to allow full growth of viable cells, thereby isolating the effect of fermentation products on inhibiting microbial growth. Media without the fermented streams were used as positive growth controls. Additionally, agar-solidified barbecue streams were used to assess whether they contained sufficient nutrients to support microbial growth.

Regarding LAB testing, general inhibition was observed across all tested microorganisms in fermented BBQ25 and BBQ50. Both fermented streams inhibited *L. parabuchneri* and *L. brevis*, as evidenced by the decrease in the dilution at which growth was observed. Higher growth inhibition was obtained for BBQ50, with no growth detected up to a 10^−1^ dilution. Similarly, no significant growth was observed in the solidified streams when the fermented stream was the sole nutrient source, confirming that the growth conditions for both microorganisms were absent only in the fermented streams.

Regarding the tested pathogenic bacterium *Bacillus cereus*, no growth was observed on solidified streams, indicating that neither of the two incorporation conditions was favorable for spore germination or microbial proliferation. The low pH levels recorded in both samples likely contributed to this inhibitory effect [89], with a pH of 4 preventing *B. cereus* spore germination at 25 °C. Environmental conditions are even harsher when associated with higher osmotic pressure in BBQ50 medium and do not allow growth or inhibit sporulation and spore germination from the first few hours of contact [90,91].

Lactic acid bacteria, which play an important role in fermentation, generate active metabolites such as organic acids—primarily lactic and acetic acid—that reduce medium pH and are toxic due to their acidic nature, as confirmed by pH measurements and HPLC analysis. Furthermore, LAB can synthesize other microbial compounds, including peptides and bacteriocins [92,93]. These results indicate that the antimicrobial metabolites produced effectively inhibit the growth of this spoilage bacterium in the fermented product.

As shown in Figure 8, before fermentation, all yeast strains grew more in the nutrient-rich YPD medium, which is expected to provide optimal conditions for microbial growth.

Overall, inhibition was observed across all yeast strains evaluated, as evidenced by a decrease in the total number of viable cells at a higher stream concentration (BBQ50). This inhibitory effect was more pronounced in *Z. rouxii* and *S. stellata*.

*Z. bailii* and *Pichia manshurica* exhibited strong growth on both YPD and agar media, showing only slight inhibition after fermentation, as indicated by a log reduction in cell viability. This result suggests that these species may exhibit high resistance to antimicrobial compounds in the barbecue stream, likely because of their ability to withstand the active metabolites produced by lactic acid bacteria [53,54,55] and the production of antimicrobial peptides by *S. boullardi* [37,94] during fermentation. Similar resistance was observed at higher levels of incorporation.

## 4. Conclusions

In conclusion, this study demonstrated that fermentation using a consortium of *S. boulardii* and the three LABs (*Lactobacillus plantarum*, *Lactobacillus rhamnosus*, and *Weissella confusa*) was successful at both incorporation levels (25% and 50%) and remained stable at room temperature without pasteurization due to antimicrobial activity.

Among the two concentrations, BBQ25 produced the most lactic acid, whereas BBQ50 generated the most ethanol. In terms of antioxidant capacity and total phenolic content, BBQ50 consistently showed higher values than BBQ25, although there were no significant differences between the unfermented and fermented samples using the FRAP and ABTS^+^ methods.

The viscosity of BBQ25 significantly increased compared to that of the unfermented sample, likely due to the exopolysaccharides produced by LAB. BBQ50 also showed a significant increase, although it was less pronounced than that of BBQ25.

Ethanol was the main metabolite produced throughout the shelf life of both fermented BBQ products. Finally, antimicrobial tests indicated that both products exhibited antimicrobial and antifungal activity against food contaminants relevant to the sauce industry that can cause spoilage. Overall, the results demonstrate that fermentation is a sustainable and viable method for adding value to BBQ waste streams, with potential as ingredients or in the development of new products.

Future research should examine the reincorporation rate of the fermented product into target products to maximize its circularity and evaluate its impact on the formulation and structure of sauces. This study lays the groundwork for potentially replacing preservatives, such as potassium sorbate, either partially or entirely and offers the possibility of substituting thickeners, such as modified starch, in sauce formulations, aligning with sustainability trends and consumer demand for clean-label products.

## Figures and Tables

**Table 1:** Food contaminants and pathogen microorganisms used to test the antimicrobial activity of BBQ25 and BBQ50.

Microorganisms	Name	Collection Number	Culture Medium
Lactic acid bacteria	*Levilactobacillus brevis*	DMS 20054	De Man, Rogosa and Sharpe (MRS)
	*Lentilactobacillus* *parabuchneri*	ISA 4389	
Yeast	*Zygosaccharomyces bailii*	ISA 1307	Yeast Extract Peptone Dextrose (YPD)
	*Zygosaccharomyces rouxii*	ISA 1770	
	*Pichia manshurica*	ISA 2426	
	*Starmerella stellata*	ISA 2339	
Pathogenic bacteria	*Bacillus cereus*	BISA 4043	Brain Heart Infusion (BHI)

**Table 2:** Concentration of sugars and primary metabolites at the beginning (NF) and end (NF) of the fermentation process of diluted barbecue steam. Values are expressed as the mean ± standard deviation (*n* = 2). All values presented for the different sugars and metabolites were significantly different, except where indicated by ns.

Metabolites ^1^ (g/100 g Product) ^2^ (v/100 g Product)	Samples		
		BBQ25	BBQ50
Glucose ^1^	NF	3.542 ± 0.260	6.553 ± 0.405
	F	0.018 ± 0.000	0.052 ± 0.001
Fructose ^1^	NF	2.922 ± 0.066	5.553 ± 0.054
	F	0.038 ± 0.038	0.198 ± 0.004
Maltotriose ^1^	NF	0.468 ± 0.001	0.560 ± 0.237
	F	0.403 ± 0.005	0.816 ± 0.086
Maltose ^1^	NF	1.293 ± 0.004	2.405 ± 0.036 ^ns^
	F	0.460 ± 0.004	1.675 ± 0.037 ^ns^
Glycerol ^1^	NF	0.026 ± 0.003	0.006 ± 0.001
	F	0.195 ± 0.003	0.399 ± 0.010
Ethanol ^2^	NF	0.005 ± 0.001	0.014 ± 0.001
	F	0.934 ± 0.007	2.127± 0.077
Lactic acid ^1^	NF	0.000 ± 0.000	0.000 ± 0.000
	F	1.285 ± 0.044	1.124 ± 0.076
Acetic acid ^1^	NF	0.160 ± 0.007 ^ns^	0.319 ± 0.006 ^ns^
	F	0.180 ± 0.000 ^ns^	0.364 ± 0.026 ^ns^

**Table 3:** Total protein and humidity content of barbecue stream diluted 1:2 (BBQ50) and 1:4 (BBQ25) before and after fermentation. Values are expressed as mean ± standard deviation (*n* = 3). Different letters indicate significant differences (*p* < 0.05) between non-fermented (NF) and fermented (F) samples for each stream concentration and each type of analysis according to a *t*-test.

Samples		Total Protein Content (% Product)	Humidity (% Product)
BBQ25	NF	0.636 ± 0.014 ^a^	79.957 ± 0.100 ^b^
	F	0.525 ± 0.027 ^b^	87.041 ± 2.22 ^a^
BBQ50	NF	1.021 ± 0.073 ^A^	89.716 ± 0.034 ^B^
	F	0.800 ± 0.022 ^B^	92.614 ± 0.364 ^A^

**Table 4:** Phenolic content of diluted barbecue samples before and after fermentation. Values are expressed as mean ± standard deviation (*n* = 3). Different letters indicate significant differences (*p* < 0.05) between non-fermented (NF) and fermented (F) samples for each stream concentration and each type of analysis according to the *t*-test.

Samples		Total Phenolic Content (mg GAE/100 g Product)	
		TPC	TPI
BBQ25	NF	13.742 ± 2.252 ^b^	9.644 ± 0.118 ^b^
	F	26.683 ± 2.252 ^a^	10.887 ± 0.033 ^a^
BBQ50	NF	37.617 ± 2.927 ^B^	16.989 ± 0.822 ^A^
	F	59.408 ± 2.061 ^A^	17.382 ± 0.383 ^A^

**Table 5:** Antioxidant power of BBQ25 and BBQ50 samples before and after fermentation, as evaluated by DPPH and FRAP assays. Different letters indicate significant differences (*p* < 0.05) between non-fermented (NF) and fermented (F) samples for each concentration and each type of analysis, according to the *t*-test.

Samples		Antioxidant Capacity (mg TEAC/100 g Product)		
		DPPH	FRAP	ABTS^+^
BBQ25	NF	10.132 ± 1.582 ^b^	67.088 ± 0.923 ^A^	111.730 ± 6.360 ^I^
	F	18.548 ± 0.670 ^a^	69.602 ± 1.925 ^A^	124.210 ± 1.272 ^I^
BBQ50	NF	15.826 ± 0.995 ^a^	146.067 ± 2.603 ^A^	99.700 ± 2.385 ^I^
	F	17.060 ± 2.200 ^a^	148.588 ±1.159 ^A^	116.789 ± 5.088 ^I^

## Data Availability

The original contributions presented in this study are included in the article. Further inquiries can be directed to the corresponding author.
